# Potential effects of sugar-sweetened beverage taxation on oral and systemic health outcomes

**DOI:** 10.3389/froh.2026.1906968

**Published:** 2026-08-12

**Authors:** Rahnuma Ahmad, Muhammad Zubeir Noorani, Mainul Haque, Mohammed S. Razzaque

**Affiliations:** 1 Department of Physiology, Medical College for Women and Hospital, Dhaka, Bangladesh; 2 Department of Medical Education, School of Medicine, University of Texas Rio Grande Valley (UTRGV), Edinburg, TX, United States; 3 Department of Pharmacology and Therapeutics, Eastern Medical College and Hospital, Cumilla, Bangladesh; 4 Global Research Fellow, Faculty of Medicine, Universiti Kuala Lumpur Royal College of Medicine Perak (UniKL RCMP), Ipoh, Perak, Malaysia

**Keywords:** dental caries, oral health, public health policy, sugar-sweetened beverages, taxation

## Sugar-sweetened beverages (SSBs)

High consumption of sugar-sweetened beverages (SSBs) has become a major global health concern. Owing to their high sugar content, particularly high-fructose corn syrup, and high caloric density, excessive intake of SSBs is a major contributor to several noncommunicable diseases (NCDs), including metabolic syndrome and cardiovascular and cerebrovascular disorders (1). Using the Global Burden of Disease Study database, Ge et al. reported that the global prevalence of high SSB consumption among young adults aged 15–39 years rose from 6.58% in 1990 to 11.13% in 2021, with projections indicating further increases through 2050 (2). Analyzing the same Global Burden of Disease database, Lara-Castor et al. found that global SSB intake among children and adolescents aged 3–19 years increased by 23% from 1990 to 2018 (3). These findings show that SSBs are being consumed more by children, adolescents, and young adults around the world, and that current efforts are not enough to reverse this trend. Because SSBs are a major source of free sugars, drinking too much of them directly causes tooth decay (the most common non-communicable disease globally) and raises the risk of obesity, type 2 diabetes, heart disease, and related conditions. The fastest growth in SSB consumption is now in low- and middle-income regions, especially sub-Saharan Africa and parts of Asia, where people often have less access to preventive dental and medical care. This makes the problem both a health and an equity issue. Stronger, population-wide measures are therefore needed, such as taxes on sugary drinks, clear warning labels, limits on marketing to children, incentives for companies to reduce sugar, and better access to safe drinking water, to cut SSB intake and reduce the burden of both oral and systemic diseases.

## SSBs, oral health, and economic burden

The association between SSB consumption and oral disease is well established. SSBs, including sodas, energy drinks, and flavored beverages, are major dietary sources of free sugars and acidity that promote oral microbial dysbiosis, enamel demineralization, and dental caries (4, 5). Their sugars are fermented by cariogenic bacteria such as *Streptococcus mutans* (6), producing organic acids that lower the oral pH and gradually dissolve tooth enamel, while their intrinsic acidity further accelerates enamel erosion and tooth decay. Multiple studies have shown a direct association between high added-sugar intake and the development and progression of periodontal disease, likely through local and systemic inflammation (7). A 2022 systematic review of 13 observational studies found that 11 reported a significant association between the frequent consumption of free-sugar-containing foods or SSBs and a higher incidence of periodontal disease (8). Similarly, an NHANES III analysis of 2,437 young adults aged 18–25 years found that higher added-sugar intake was significantly associated with an increased risk of periodontal disease (9). In addition, a recent systematic review and meta-analysis showed that reducing free-sugar intake significantly improved gingival health and reduced gingival inflammation, further supporting the inflammatory role of excessive sugar consumption in periodontal disease progression (10). Therefore, SSBs contribute to oral disease through both cariogenic and erosive mechanisms: their fermentable sugars, especially sucrose, promote acid production by dental biofilms, while their intrinsic acidity increases the risk of enamel erosion (11).

The global economic burden of oral diseases is estimated to exceed US$710 billion annually, including approximately US$387 billion in direct treatment costs and US$323 billion in productivity losses, highlighting their major public health and socioeconomic impact (12). Oral diseases also significantly reduce quality of life and overall well-being. Direct oral healthcare costs vary widely, from about US$0.52 per capita in lower-income countries to roughly US$260 per capita in high-income countries (12). Dental caries is among the most common NCDs worldwide; according to the WHO, more than 530 million children have caries in primary teeth, and untreated caries in permanent teeth affect an estimated 2.4 billion people globally (13). Although highly preventable, dental caries remains a major public health challenge because of its effects on systemic health, its disproportionate burden on disadvantaged groups, and its substantial treatment costs (14). Limited access to oral healthcare also leads to large losses in productivity and human capital, often captured as indirect costs through absenteeism and presenteeism. These effects are reflected in higher DALYs and YLDs, underscoring the broad human and economic costs of oral disease (15).

## Taxation of SSBs

**Figure 1:**
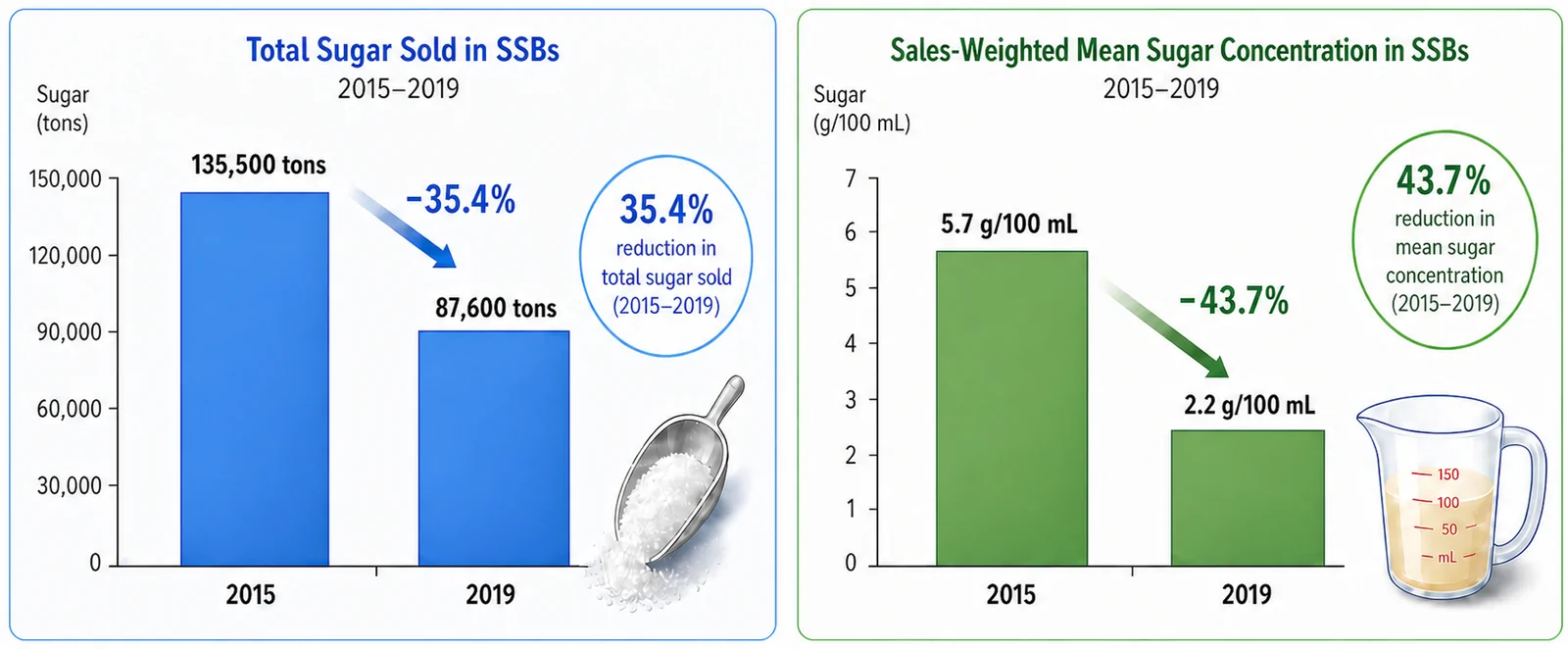
The soft drinks industry levy (SDIL), introduced in the United Kingdom in 2018, has had a substantial effect on soft drink reformulation and market sales. Since its implementation, many beverages have contained less sugar, and sales of sugar-sweetened beverages (SSBs) have declined. According to Public Health England's progress report covering 2015–2019, total sugar sales from soft drinks decreased by 35.4%, from 135,500 tons in 2015 to 87,600 tons in 2019. Over the same period, the average sugar content of soft drinks (weighted by how much each drink sold) fell from 5.7 g to 2.2 g per 100 mL. Public Health England also reports a separate figure of −43.7%, which is an estimate of how much sugar reduction can be attributed specifically to the Soft Drinks Industry Levy. This number comes from a detailed modelling approach and is not simply the percentage drop that would be calculated from the average sugar values alone (18).

Increasing fiscal taxation on SSBs constitutes an evidence-based public health intervention. The underlying mechanistic rationale is that price increases at the upper end of the price distribution are likely to induce shifts in consumer dietary behavior, reducing the consumption of high-calorie, low-nutrient foods and SSBs. Consequently, this approach reduces the prevalence of lifestyle- and nutrition-related NCDs, such as obesity and type 2 diabetes mellitus, while simultaneously generating fiscal resources that can be allocated to public health programs. Cárdenas-Torres et al. reported that policies that substantially increased taxes on SSBs successfully reduced the consumption of SSBs (16). However, ideological debates and political opposition, particularly strong industry lobbying, concerns about regressive impacts on low-income populations, and voter resistance to new taxes, have created significant challenges for advancing the taxation of SSBs on policy agendas and securing legislative approval worldwide (16). Despite these barriers, several countries have successfully implemented policy strategies to overcome regulatory challenges. For instance, Mexico earmarked tax revenue on SSBs to fund free drinking water in schools and public health programs, while the United Kingdom paired its Soft Drinks Industry Levy with transparency requirements and industry collaboration to encourage reformulation. In Brazil, framing the taxation of SSBs as part of a broader food security and child health agenda helped develop public support and reduce industry pushback. These examples illustrate how strategic framing, revenue earmarking, and multisectoral partnerships can enhance the political feasibility of the taxation of SSBs across diverse contexts. Soft Drinks Industry Levy, introduced in the United Kingdom in 2018, implements a tiered excise tax linked to sugar content. This policy has compelled soft drink manufacturers to reformulate their products by removing added sugar from retail shelves. One year after the implementation of the UK Soft Drinks Industry Levy, free sugar consumption decreased significantly among both children and adults. Daily free sugar intake from soft drinks decreased by approximately 3 g/day in children and 5 g/day in adults, indicating a beneficial effect on dietary sugar intake (17). It has been reported that total sugar sales from drinks decreased by 35.4%, from 135,500 tons in 2015 to 87,600 tons in 2019 (18). During the same period, the sales weighed average total sugar content decreased from 5.7 g per 100 mL in 2015 to 2.2 g per 100 mL in 2019, which is a decrease of 43.7% (Figure 1) (18). It should be noted that the −43.7% figure is Public Health England's Soft Drinks Industry Levy-specific, modelled reduction metric, not the simple percentage change in the sales-weighted average; of relevance, the arithmetic drop from 5.7 g to 2.2 g per 100 mL corresponds to approximately 61%. In Mexico, an 8% ad valorem tax on nonessential energy-dense foods (NEDFs), including sweetened baked goods, candies, snacks, and foods exceeding 275 kcal per 100 g, is imposed (19). Of relevance, SSBs are excluded from the 8% NEDF tax; instead, they are subject to their own excise tax on volume. This policy provides a useful example for other countries, suggesting that taxing high-calorie, low-nutrient ultra-processed foods may support healthier dietary choices and public health (19). Taxes on SSBs are typically designed as 1) excise taxes, which are levied on the producer or importer and often passed on to consumers through higher shelf prices, or 2) tiered sugar-content taxes, which impose higher rates on drinks with greater sugar concentrations, or 3) volume-based taxes, in which the tax depends on the quantity of SSBs sold (e.g., per ounce or per liter), meaning that larger-volume drinks are taxed more heavily regardless of their sugar concentration. In 2014, Mexico introduced a 1-peso-per-liter excise tax on SSBs. Relative to pretax trends, purchases of taxed SSBs decreased by approximately 5.5%–6% in 2014, with this reduction further increasing to 9.7%–12% in 2015. This progressive decline suggests the impact of taxes over time and supports their potential effectiveness as a policy intervention for reducing SSB consumption (20). South Africa implemented volume-based Health Promotion Levy in 2018. Limited evidence suggests that the tax may have reduced added sugar consumption, and as per the Republic of South Africa, National Treasury, the levy generated approximately R3.2 billion (≈US$228 million) in revenue in the fiscal year (2018/19), while observational studies indicate reductions in purchases of taxed beverages and industry reformulation toward lower-sugar products (21). The WHO frequently recommends that revenues from taxes on SSBs be earmarked exclusively for public health initiatives addressing disorders related to the consumption of SSBs; however, national authorities often allocate these additional government revenues to general budgetary purposes instead.

## Limited oral health focus in SSB taxation

Research on the taxation of SSBs has focused predominantly on multisystemic health outcomes, including obesity, heart failure, hypertension, metabolic syndrome, and type 2 diabetes mellitus. However, minimal attention has been given to the effects of the taxation of SSBs on general oral and dental health, particularly among children, adolescents, and individuals from low-income communities. Key oral health conditions such as dental caries (tooth decay) and dental erosion (enamel demineralization) remain critically under investigation in the literature on the taxation of SSBs. A recent review revealed only five eligible studies assessing oral health-related outcomes, and all five were modeling studies, not real-world evaluations (22). In a modeling study in Australia, Sowa et al. projected that an SSB tax could improve oral health by reducing dental caries and dental treatment costs, with the greatest benefits expected in younger age groups (23). In a simulation study, Urwannachotima et al. projected that an SSB tax alone would have only a modest effect on dental caries in Thailand, with a reduction in prevalence of approximately 1% by 2040, whereas a more aggressive policy could reduce it by about 21% (24). Available studies suggest that oral health evidence remains sparse, with only a small number of reviews and a limited direct evidence base for dental endpoints (22, 25). The results of the Berkeley evaluation suggest that oral health was acknowledged in the debate on the taxation of SSBs but was not a central or strongly mobilized theme. Although oral health was identified as an important public health issue, it remained underused in campaign messaging, and dental professionals were not prominently involved in the advocacy effort. In the communication materials analyzed, oral health and tooth decay appeared only rarely, indicating that the debate was shaped more strongly by broader concerns such as obesity, diabetes, and general public health than by dental outcomes (26). Of relevance, three years after the implementation of Berkeley's SSB tax, residents were consuming substantially fewer sugary beverages (21% reduced in Berkeley) and more water (63% increase in Berkeley), with these changes sustained relative to nearby cities without such a tax (27, 28). Again, Berkeley SSB tax evaluations have largely focused on prices, sales, consumption, and obesity-related outcomes, and have not reported dental endpoints as measured outcomes (26). In their systematic review and meta-analysis, Andreyeva et al. reported that implemented SSB taxes were associated with higher prices of taxed beverages and lower sales, with an estimated 82% pass-through and a 15% decline in SSB sales. This review examined consumption, diet, body weight, product changes, unintended consequences, health, and pregnancy outcomes but did not evaluate oral health outcomes (29). In a similar line of study, Falbe et al. summarized the population-health effects of SSB taxes, emphasizing reductions in beverage consumption and potential improvements in weight-related outcomes rather than oral health endpoints (30). Wang et al. modeled a penny-per-ounce excise (POE) tax and estimated effects on SSB consumption and chronic disease outcomes, including diabetes and cardiovascular events, as well as health spending; oral health outcomes were not included in the model (31). This represents a substantial gap not only in dental care research but also in population health and social health economics, given that dental caries is the most prevalent NCD globally and that oral diseases impose over $710 billion in annual economic burden worldwide (12). Furthermore, there is a substantial lack of public awareness regarding both the harmful health effects of SSBs and the active efforts aimed at reducing their consumption. Although societal awareness of the harm that added sugar causes to oral health is generally high, strategies for the taxation of SSBs frequently encounter implementation and behavioral challenges (16). Addressing this knowledge gap regarding the harmful health impacts of SSBs and the rationale for taxation policies requires stronger, targeted educational campaigns to alleviate these obstacles. Both volumetric and tiered sugar taxes have been identified as effective models for reducing oral and dental diseases by discouraging the consumption of SSBs through additional taxation. A 20% volumetric tax on SSBs was estimated to reduce free sugar intake by approximately 4.4 g/day in high-income countries and 4.0 g/day in low- and middle-income countries; reduced sugar intake was associated with modest but measurable improvements in oral health, including a lower prevalence of dental caries in children and slight reductions in caries severity among adults over a 10-year period (25). Using a tooth-level Markov model, Jevdjevic et al. evaluated the potential impact of a 20% tax on sales of SSBs in the Netherlands; the model projected a gain of 2.13 caries-free tooth-years per individual, with boys aged 6–12 years experiencing the greatest benefit (32). Although the taxation of SSBs has been associated with a reduction in dental caries and dental care costs, most existing evidence is derived from modeling and simulation studies, highlighting the need for more real-world observational and longitudinal studies (33). The minimal evidence linking policies on the taxation of SSBs to oral health outcomes limits the ability of policymakers to fully appreciate the comprehensive public health benefits of fiscal interventions, particularly for vulnerable low-income people who face a disproportionate burden of both oral disease and related health risks with the consumption of SSBs.

## Effects of the taxation of SSBs on NCDs

As previously discussed, fiscal policies are key instruments used by governments to generate revenue, allocate resources, and regulate economic activity to curb the rising incidence of NCDs, such as type 2 diabetes mellitus, cardiovascular disease, and obesity, while also helping to offset increasing healthcare expenditures. Meta-analytic evidence from 33 high-quality studies across 16 jurisdictions demonstrates that taxes on SSBs are associated with an average 15% reduction in sugary drink sales (95% CI: −20% to −9%) (29, 34). The price elasticity of demand for SSBs is approximately −1.59 (95% CI: −2.11 to −1.08), indicating that a 10% price increase would reduce consumption by approximately 16%. In separate analyses, a 10% tax on SSBs has been associated with an average decrease in purchases and dietary intake of approximately 10% (95% CI: −5.0% to −14.7%), although the observed effect depends on the tax pass-through and the study setting (29). Additionally, the UK Soft Drinks Industry Levy was associated with an 8% relative reduction in obesity among girls aged 10–11 years (equivalent to preventing 5,234 cases annually in this group) (35), and tax in Mexico was linked to a 1.3% reduction in overweight/obesity prevalence among adolescent girls (36). These taxes also drive product reformulation toward lower sugar content. Studies have shown that children, adolescents, and young adults are particularly responsive to increases in the price of SSBs, leading to marked reductions in purchases and sustained declines in the prevalence of obesity among children (37). U.S.-based modeling studies suggest that a nationwide POE tax on SSBs could substantially reduce incident type 2 diabetes and may outperform the National Diabetes Prevention Program (DPP) in population-level cost-effectiveness and impact. In a head-to-head comparison, compared with the National DPP, the POE tax was projected to prevent more diabetes and generate greater cost savings per case (38). Wang et al. estimated that a POE tax would reduce SSB consumption by 15% among adults aged 25–64 and would reduce new diabetes cases by 2.6% overall over 10 years (31). The investigators recommend that implementing the POE tax alongside the DPP may provide complementary benefits and could enhance diabetes prevention and cost savings over time. These substantial systemic benefits provide a useful precedent for oral health modeling, in which similar tax-related reductions in sugar intake may yield meaningful gains in caries prevention and other oral health outcomes.

## Challenges and unintended consequences of taxation on SSBs

Taxation of SSBs may be regressive, as low-income households can share a larger proportion of their income with the tax despite paying relatively small absolute amounts. This concern is especially salient because sugary drinks are often more affordable and more frequently consumed in disadvantaged populations, even though these groups also carry a greater burden of diet-related disease. As a result, the equity implications are complex: the financial burden may fall disproportionately on low-income households, while the potential health benefits may also be greater in the same groups (39, 40).

Another unintended consequence is substitution, where consumers reduce taxed beverage intake but shift toward untaxed sugary foods or other caloric products. This weakens the intended health effect because lower consumption of SSBs does not necessarily translate into lower overall sugar or calorie intake. Evidence from local tax evaluations has been mixed: some settings show little substitution, while others show increases in sweets or other untaxed items. Therefore, the tax may successfully change beverage purchases but still fail to produce a large net improvement in diet quality if people simply move their sugar intake elsewhere. Industry responses to the taxation of SSBs often include product reformulation through the substitution of sugar with nonnutritive sweeteners. Although this may lower the added sugar content, it may also limit the public health impact of the policy if reformulated products continue to reinforce sweet taste preferences or if the long-term health implications of increased non-nutritive sweetener exposure remain uncertain.

**Figure 2:**
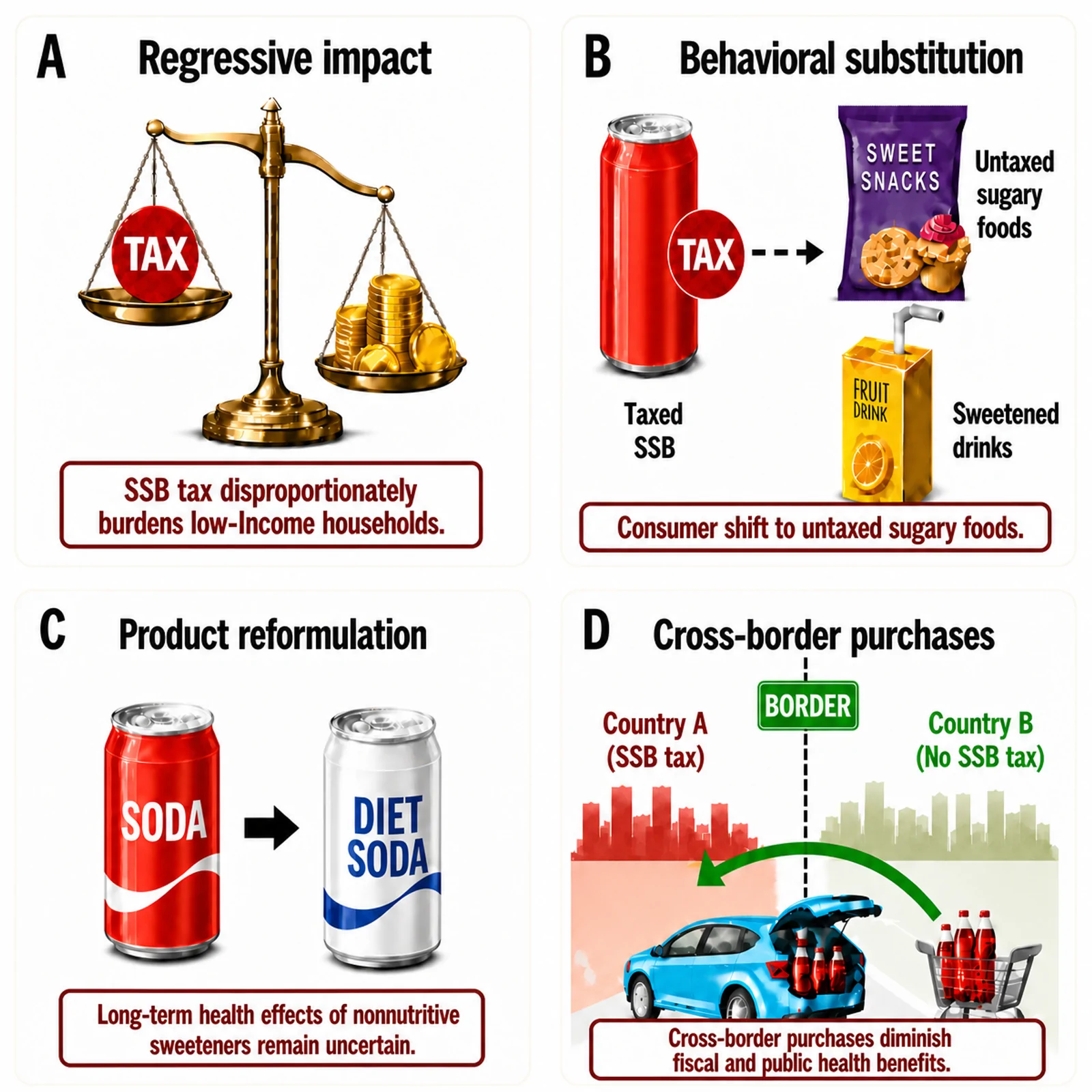
Limitations of tax on sugar-sweetened beverages (SSBs): taxes on SSBs can be regressive, disproportionately affecting low-income households and spending a greater proportion of their income on taxed beverages. Consumers may substitute taxed beverages with untaxed sugary foods or drinks, reducing the intended health benefits. Manufacturers may reformulate products with nonnutritive sweeteners, whose long-term health effects remain uncertain while preserving preferences for sweet tastes. Cross-border purchases may further reduce tax effectiveness. In addition, differences in culture, baseline sugar consumption, and policy design and enforcement contribute to heterogeneous outcomes across countries (39–42).

Cross-border purchasing represents an important limitation of the taxation of SSBs, particularly in adjacent taxed and untaxed jurisdictions. When consumers can easily purchase beverages outside the taxed area, the policy effect may be diluted because consumption is displaced rather than eliminated. Such avoidance may also raise equity concerns, as households with greater mobility are better able to evade the tax. This issue is especially relevant for local and city-level taxes, where geographic leakage can substantially undermine population-level impact (41). The impact of the taxation of SSBs is not uniform across countries because consumer behavior, tax design, enforcement capacity, market structure, and baseline diet patterns differ substantially. Consequently, a tax that performs well in one setting may have weaker effects elsewhere if the pass-through is incomplete, substitution is easier, or implementation capacity is limited. This heterogeneity means that findings from one country should not be generalized uncritically to another. It also helps explain why the same policy may appear strongly effective in one evaluation but only modestly effective in another since outcomes are shaped by local purchasing habits, retail environments, and enforcement conditions (Figure 2) (42). For instance, in a real-world evaluation of 83,260 dental patients, the Philadelphia beverage tax was not associated with reduced tooth decay in the overall population, but it was associated with lower new decay, missing, and filled teeth among Medicaid patients (generally represent lower-income patients) (43).

## Conclusion

Globally, SSB consumption is a well-established contributor to childhood obesity and adverse oral health outcomes; nevertheless, SSB intake continues to rise worldwide (44). Although SSB taxation is increasingly recognized as a policy with potential oral health benefits, oral outcomes remain insufficiently incorporated into evaluation frameworks. This omission likely limits the apparent value of these interventions, since dental caries and periodontal disease are common, preventable, and unequally distributed. Future assessments should therefore include oral health endpoints alongside systemic and economic outcomes to provide a more complete estimate of policy impact.
